# Kimura disease presenting as a right lower limb soft tissue tumor: a case report

**DOI:** 10.3389/fmed.2026.1838288

**Published:** 2026-06-22

**Authors:** Kun Hao, Feng Gao

**Affiliations:** Department of Pathology, Hebei Medical University Third Hospital, Shijiazhuang, China

**Keywords:** eosinophil, Kimura disease, limbs, pathology, soft tissue tumor

## Abstract

Kimura disease (KD) is a rare chronic inflammatory disorder. Its pathogenesis remains unclear but is believed to involve dysregulated immune responses. It is characterized by soft tissue masses in the head and neck, elevated eosinophil counts in peripheral blood, and elevated serum IgE levels. This case report describes a 53-year-old Asian male who presented with a localized mass on the anterior aspect of the right lower leg without abnormalities in peripheral blood. Additionally, eight articles published between 2015 and 2025 were reviewed, summarizing 12 patient cases to identify common aspects in diagnosing and treating KD, primarily characterized by limb lesions.

## 1 Introduction

Kimura disease (KD) is a rare, benign, chronic inflammatory disorder of uncertain etiology, first described in the Chinese literature in 1937 under the designation “eosinophilic hyperplastic lymphogranuloma,” and subsequently characterized and named by Kimura and colleagues in 1948 (1). The condition is pathologically defined by a triad of follicular lymphoid hyperplasia, prominent eosinophilic infiltration with microabscess formation, and small vessel proliferation within subcutaneous or glandular tissues (2, 3).

Clinically, KD most commonly manifests as painless soft tissue masses in the head and neck region, frequently involving the parotid gland, periauricular soft tissues, and cervical lymph nodes (4). Associated laboratory findings include peripheral eosinophilia and markedly elevated serum IgE levels, both of which reflect underlying Th2-skewed immune dysregulation (3). Renal involvement, predominantly in the form of nephrotic syndrome, occurs in approximately 20% of patients (5).

Although KD has well-established clinicopathological features in its typical head-and-neck presentation, involvement of the extremities remains distinctly uncommon and frequently leads to diagnostic uncertainty. Cases arising in the upper or lower limbs may lack the classical serological abnormalities, and the histopathological differential diagnosis from angiolymphoid hyperplasia with eosinophilia (ALHE) and other eosinophilic soft tissue disorders remains challenging. To date, only a limited number of extremity-predominant KD cases have been systematically reviewed.

Here, we present a case of KD manifesting exclusively as a localized pretibial soft tissue mass with normal peripheral blood eosinophil count and serum IgE, and provide a systematic review of published extremity-predominant KD cases from the past decade. The aims of this report are to characterize the clinical, radiological, and histopathological features of KD presenting in atypical anatomical sites, to emphasize the critical role of pathological examination, and to discuss the therapeutic implications for this rare presentation.

## 2 Case report

The patient is a 53-year-old Asian male with no prior medical history and no previous episodes of soft tissue swelling. He presented to our hospital 3 months after first noticing a gradually enlarging, non-painful mass at the anterior border of the right tibia. No history of local trauma, insect bite, or known allergen exposure was elicited.

**Figure 1:**
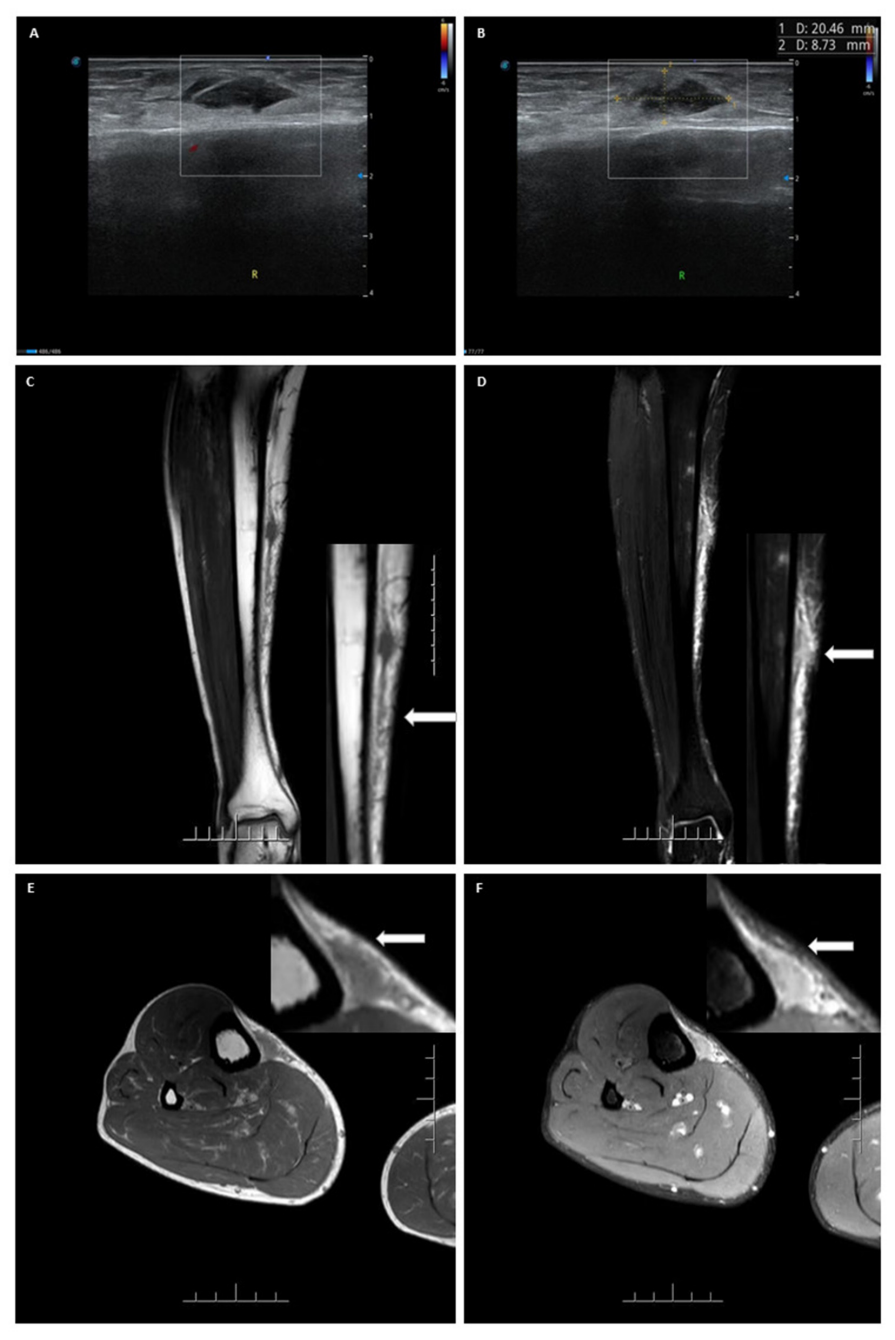
**(A, B)** Ultrasound (B-mode and CDFI) demonstrating a solid, well-circumscribed subcutaneous mass in the right anterior tibial region measuring 2.0 × 0.9 cm, with internal star-shaped vascularity on color Doppler flow imaging. Magnetic resonance imaging of the right lower leg demonstrating the subcutaneous pretibial nodule. **(C)** T1-sagittal image: the nodule (arrowhead) measures approximately 1.6 × 0.9 cm in cross-section and demonstrates signal intensity mildly higher than that of adjacent skeletal muscle, with relatively defined margins on this sequence. **(D)** T2 fat-suppressed sagittal image: the nodule (arrowhead) shows markedly increased signal intensity with ill-defined peripheral margins and adjacent soft tissue edema, consistent with peritumoral inflammatory infiltration. **(E)** T2-weighted sequence, axial plane. **(F)** T2 fat-suppressed axial image: the longitudinal extent of the nodule (arrowhead, 0.8 cm in the craniocaudal dimension) is depicted with surrounding soft tissue signal abnormality. The lesion is located in the subcutaneous compartment superficial to the deep fascia, without involvement of the underlying periosteum, tibial cortex, or medullary cavity.

Physical examination revealed a spindle-shaped mass at the anterior border of the right tibia, approximately 2 cm in maximum dimension, of moderate firmness and without tenderness or overlying skin changes. Regional lymphadenopathy was absent. B-mode ultrasonography demonstrated a solid, well-circumscribed mass measuring 2.0 × 0.9 cm within the subcutaneous fat layer at the right anterior tibial region, with heterogeneous internal echogenicity. Color Doppler flow imaging (CDFI) revealed internal stellate vascularity, consistent with intralesional vascular proliferation. Magnetic resonance imaging (MRI) of the right lower leg was performed on a 3.0-Tesla system, including axial and sagittal T1-weighted and fat-suppressed T2-weighted sequences. A subcutaneous nodule measuring 1.6 × 0.9 × 0.8 cm was identified in the right pretibial subcutaneous layer. On T1-weighted images, the nodule demonstrated signal intensity mildly higher than that of adjacent skeletal muscle. On fat-suppressed T2-weighted sequences, the nodule showed markedly increased signal intensity with well-defined margin of the mass margins, accompanied by adjacent soft tissue edema consistent with peritumoral inflammatory infiltration, while the entire lesion presents as ill-defined. The lesion was entirely confined to the subcutaneous compartment, superficial to the deep fascia, with no involvement of the underlying periosteum, tibial cortex, or medullary cavity (6, 7). No pathologically enlarged regional lymph nodes were identified (Figure 1).

Laboratory investigations, including complete blood count with differential (Eosinophil count: 0.16 × 10^9^/L, reference range: 0.02–0.52 × 10^9^/L; Eosinophil percentage: 2.9%, reference range: 0.4–8%), serum IgE (164IU/ml, reference range: 1–190IU/ml), coagulation profile and kidney function, were all within normal reference ranges and urine protein test was negative. The absence of peripheral eosinophilia and elevated IgE, while atypical, has been documented in early or localized KD presentations.

**Figure 2:**
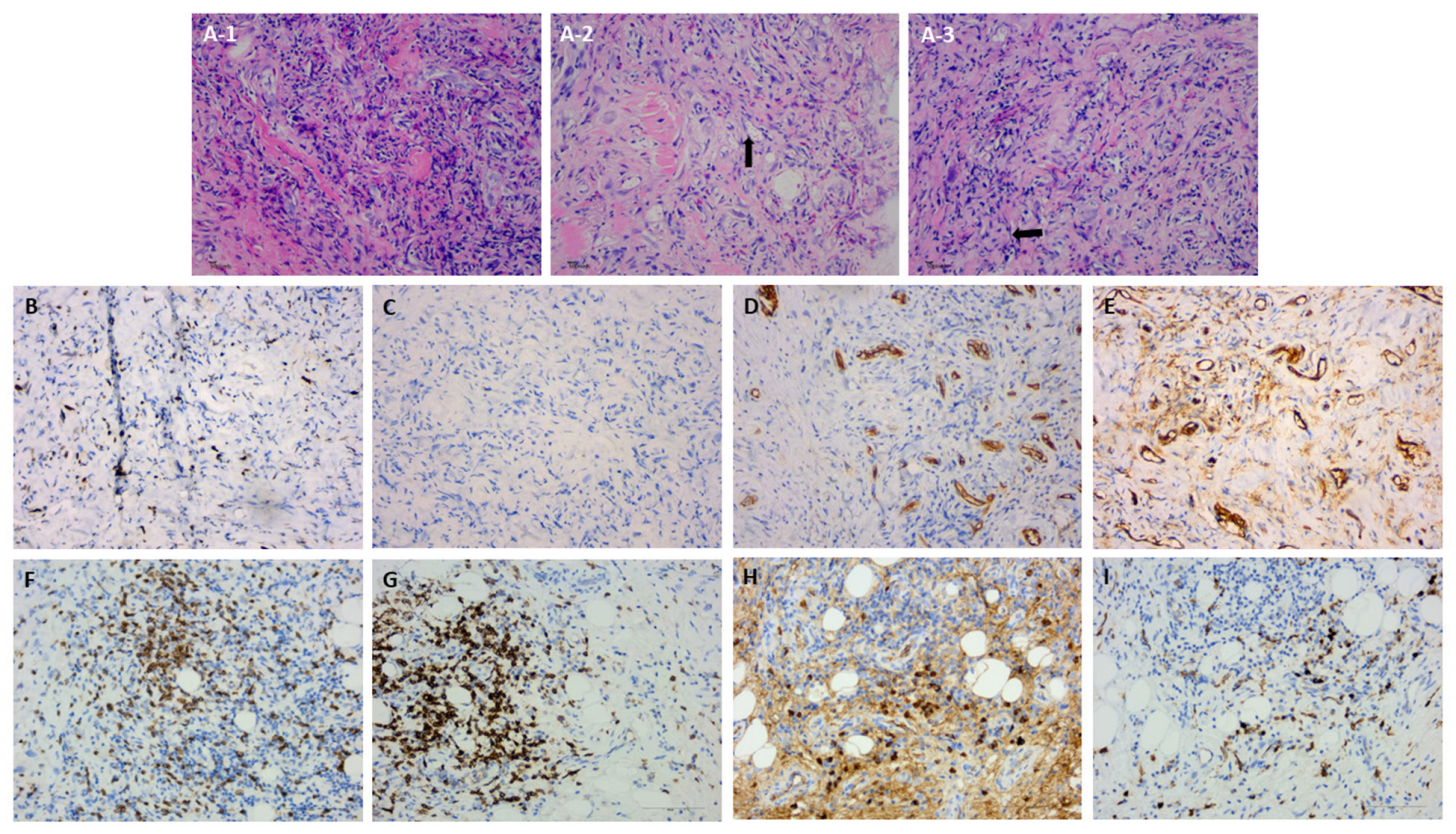
Histopathological and immunohistochemical findings. **(A)** H&E section ( × 200): Eosinophilic infiltration, small vessel proliferation and focal fibrosis. **(B)** Ki-67 immunostaining ( × 200): hotspot area ≤ 5% positive, consistent with a benign process. **(C)** CK immunostaining ( × 200): negative, excluding epithelial neoplasm. **(D)** CD31 immunostaining ( × 200): positive, demonstrating intratumoral small vessel proliferation, a recognized histological feature of KD. **(E)** CD34 immunostaining ( × 200): vascular pattern positive, corroborating small vessel proliferation within the lesion. **(F, G)** CD3 and CD20 immunostaining ( × 200): T cells and B cells are positive. **(H, I)** IgG and IgG4 ( × 200): IgG4-positive plasma cells account for less than 10% of all plasma cells.

**Figure 3:**
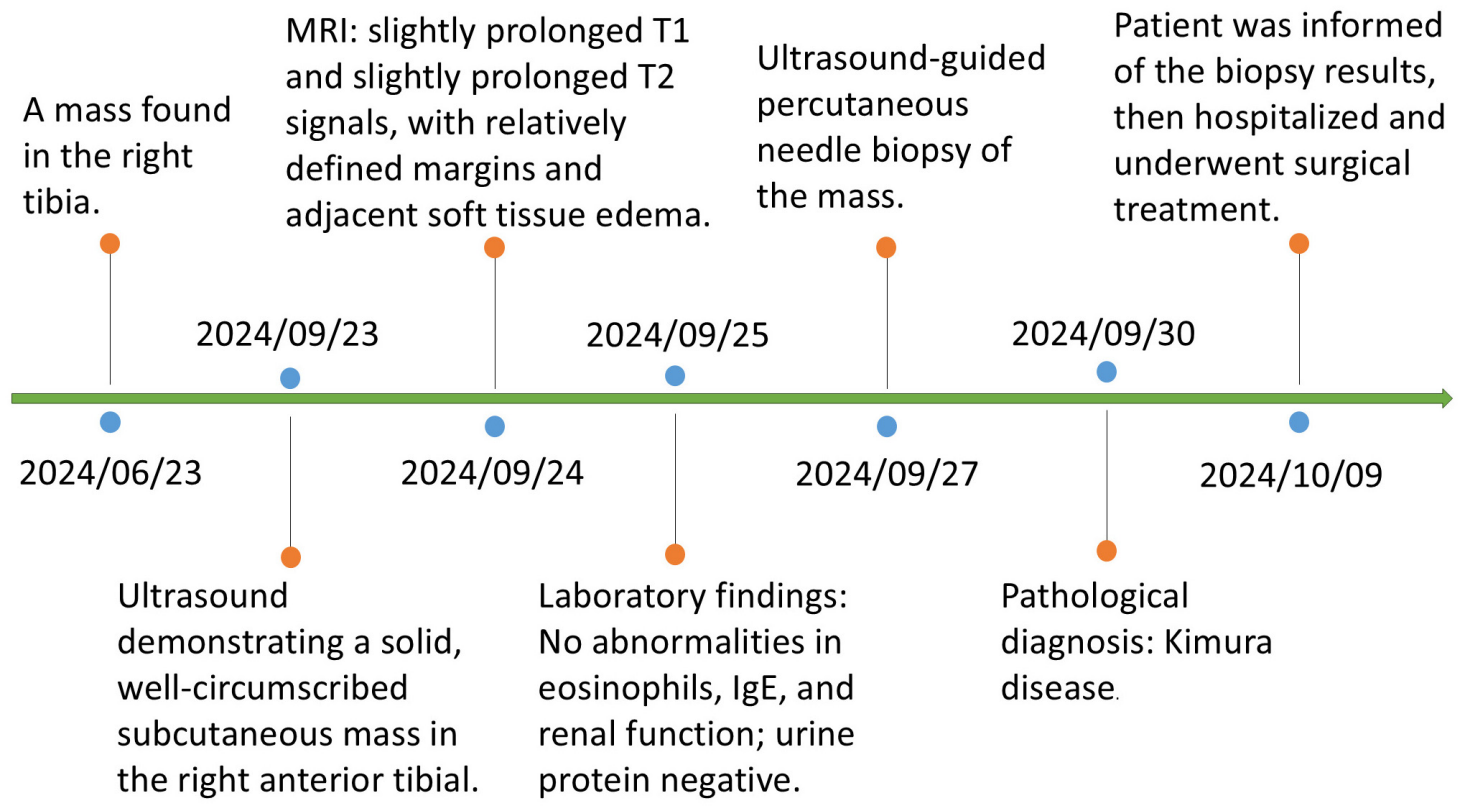
Representative timelines of patient relevant data history.

Given the indeterminate imaging characteristics, an incision biopsy was performed under ultrasound guidance on September 27, 2024, following written informed consent. Microscopic examination of hematoxylin and eosin (H&E)-stained sections demonstrated the following characteristic features: (1) lymphocyte proliferation with mature lymphocytes (Figures 2F–I represent immunohistochemical staining performed on lymphocyte-rich regions); (2) dense eosinophilic infiltration (Figure 2A−1); (3) small vessel proliferation (Figure 2A−2); and (4) focal interstitial fibrosis (Figure 2A−3). These four features collectively fulfill the established histopathological diagnostic criteria for KD (3, 6).

Immunohistochemical analysis was performed to characterize the vascular component and to exclude important differential diagnoses. CD31 and CD34 highlighted numerous small vessels throughout the lesion, confirming intratumoral vascular proliferation—a recognized histological hallmark of KD. Critically, no epithelioid or hobnail endothelial morphology was identified, arguing against angiolymphoid hyperplasia with eosinophilia (ALHE). The lymphoid infiltrate comprised a mixture of CD3-positive T cells and CD20-positive B cells, supporting a reactive polyclonal process and excluding lymphoma. IgG4 immunostaining demonstrated fewer than 10 IgG4-positive plasma cells per high-power field, with an IgG4/IgG ratio below 40%, rendering IgG4-related disease unlikely. Cytokeratin (CK) immunostaining was negative, excluding an epithelial neoplasm. Ki-67 hotspot proliferation index was less than 5%, consistent with a benign process (Figure 2). The absence of necrotizing vasculitis excluded eosinophilic granulomatosis with polyangiitis (EGPA). The integrated clinicopathological findings were conclusive for KD.

The diagnosis of KD was confirmed on the basis of integrated clinicopathological criteria. Complete surgical excision of the mass was subsequently performed. Intraoperative findings confirmed a well-encapsulated subcutaneous nodule; final pathological assessment of the resection specimen confirmed negative surgical margins. No multifocal lesions, lymph node involvement, or renal disease were identified.

Regarding postoperative management, the patient was counseled regarding the risk of local recurrence following surgical excision alone, which has been reported in 25–40% of surgically managed KD cases (8). He declined adjuvant immunosuppressive agents including cyclosporine and methotrexate due to concerns about their long-term toxicity profiles. Following shared decision-making, long-term low-dose oral glucocorticoid therapy (prednisolone 5 mg/day) was initiated as maintenance treatment. The dose was maintained at 5 mg/day throughout the follow-up period, with no dose adjustment required. No significant glucocorticoid-related adverse effects, including hyperglycemia, osteopenia, or adrenal suppression, were documented during this period. The rationale for this therapeutic approach is discussed in Section 3.6.

The patient has been followed clinically and radiologically at regular intervals for a total of 16 months, with no clinical or imaging evidence of local recurrence or new lesion development (Figure 3).

## 3 Literature review methods

A systematic literature search was conducted in PubMed and Web of Science databases, covering publications from January 2015 to April 2025. The following search terms were used: (“Kimura disease” OR “Kimura's disease” OR “eosinophilic hyperplastic lymphogranuloma”) AND (“extremity” OR “limb” OR “arm” OR “leg” OR “upper limb” OR “lower limb” OR “thigh” OR “tibia”). The search was limited to publications in English.

Inclusion criteria were: (1) Case reports or case series published between 2015 and 2025; (2) Histopathologically confirmed diagnosis of KD; (3) Primary clinical presentation attributable to a soft tissue mass of the extremity; and (4) Sufficient clinical, imaging, or pathological detail for data extraction. Exclusion criteria were: (1) Cases in which extremity involvement was secondary to a primary head-and-neck lesion; (2) Cases involving only isolated lymph node enlargement without an adjacent soft tissue mass; and (3) Cases with involvement of long bone cortex or medullary cavity.

Data were extracted independently by authors and discrepancies were resolved by consensus. Variables extracted included: nationality, publication year, patient sex and age, lesion site, imaging findings, clinical presentation, histopathological features, treatment modality, and clinical outcome. The extracted data are summarized in Table 1. This systematic review followed the PRISMA 2020 reporting guidelines. A total of eight studies encompassing 12 cases met the inclusion criteria and were included in the final analysis (Table 1).

**Table 1:** Clinical summary of reported KD cases with primary extremity involvement.

Case no.	Nationality [Ref]	Year	Sex	Age	Site	Imaging findings	Clinical presentation	Pathology	Treatment & Outcome
1	Chinese (25)	2015	M	45	Both upper limbs	MRI: Mass intensity similar to or slightly higher than muscle on T1–weighted images; intermediate complex signal on T2.	Painless swelling of both upper limbs.	Fibrofatty connective tissue with prominent lymphoid reaction, occasional atretic but intact germinal centers, and significant eosinophilic infiltration.	Surgical excision; no recurrence reported.
2	Indian (26)	2016	M	35	Right thigh & right auricle	CT: Diffuse vascular swelling within muscle, containing small lymph nodes.	Swelling in right auricle and right thigh for 2 years, with gradual enlargement.	Follicular hyperplasia with reactive germinal centers, eosinophilic folliculolysis, and eosinophilic infiltration of germinal centers.	Surgery; no recurrence on close follow–up.
3	Chinese (27)	2018	M	24 (initial); 39 (recurrence)^*^	Left upper limb	MRI: Mass intensity similar to or slightly higher than muscle on T1; high intensity on T2.	Painless mass in left upper limb; recurrence 15 years after initial excision.	Dense infiltration of eosinophils, lymphocytes and small–vessel–engorged histiocytes, along with mild fibrosis.	Surgical excision; recurrence noted.
4	Indian (28)	2022	M	20+	Medial distal left arm	Ultrasound: Mixed echogenicity mass with nodular hypoechoic lymph nodes; MRI: Hyperintense tissue on T2 with fatty septa.	Mass on medial distal left arm; history of nephrotic syndrome.	Multiple lymphoid follicles with interfollicular vascular proliferation; abundant eosinophils and scattered plasma cells.	Surgery; postoperative low–dose steroids with no recurrence.
5	Japanese (29)	2023	M	69	Right upper arm	MRI: Soft tissue mass in right upper limb; intermediate to high signal intensity on T2.	Asymptomatic swelling in right upper arm.	Lymphoid tissue with follicles and eosinophilic infiltration.	Surgical excision (wide resection); no recurrence.
6	Chinese (15)	2024	M	5–34 (multiple cases)	Upper arm	CT: Single or multiple isodense nodules; MRI: Isointense on T1, slightly high on T2, with perilesional edema and thinning of regional subcutaneous fat.	Single or multiple masses in the upper arm.	Lymphoid hyperplasia, eosinophilic infiltration, and high endothelial venule proliferation.	Surgical excision; outcomes not fully reported.
7	Chinese (30)	2025	F	16	Right lower limb	Not available.	Pruritic plaque on right lower limb, enlarged after scratching.	Dense dermal infiltration of lymphocytes and scattered eosinophils, follicular–like structures in deep dermis, and mild small vessel proliferation.	Surgical excision; no signs of recurrence postoperatively.
8 (present)	Chinese	2024	M	53	Right lower leg (pretibial)	Ultrasound: Solid mass 2.0 × 0.9 cm, subcutaneous, star–shaped blood flow on CDFI. MRI: Subcutaneous nodule 1.6 × 0.9 × 0.8 cm, isointense on T1, mildly hyperintense on T2, adjacent soft tissue edema.	Painless spindle–shaped mass at right pretibial region; normal peripheral blood eosinophils and serum IgE.	Follicular hyperplasia, eosinophilic infiltration, local fibrosis; CD31(+), CD34(vascular+), CK(–), Ki−67 < 5%, IgG4/IgG ratio below 40%. Consistent with KD.	Surgical excision + long–term low–dose glucocorticoid; no recurrence at 16 months.

## 4 Discussion

### 4.1 Epidemiology

KD is a rare chronic inflammatory disorder. First described in 1937 by Chinese researchers, it was initially termed “eosinophilic hyperplastic lymphogranuloma.” Japanese researchers subsequently clarified its pathological characteristics and named it “Kimura disease (KD)” in 1948 (1). KD shows clear predilections related to sex, age, and geography. Epidemiological data indicate the disease predominantly affects males, with a male-to-female ratio as high as 19:1 (9). The disease typically manifests during the second and third decades of life (10). Although significantly more prevalent in Asian regions, including Japan, China, and Indonesia (11), sporadic cases have been reported in Europe and the Americas. Among the 12 reviewed cases, all originated from Asian countries (China, India, Japan), and males comprised the majority (11/12), consistent with established epidemiological patterns.

### 4.2 Pathophysiology

The pathogenesis of KD remains incompletely understood, but is strongly implicated in Th2-mediated immune dysregulation. Elevated levels of Th2-associated cytokines—including interleukin (IL)-4, IL-5, and IL-13—have been consistently demonstrated in KD patients. These cytokines promote IgE secretion from B cells and facilitate recruitment, activation, and prolonged survival of eosinophils. Eosinophils contribute to tissue injury and progressive fibrosis through the release of granular proteins such as eosinophil cationic protein (ECP) and eosinophil-derived neurotoxin (EDN). Moreover effector cells, including mast cells, basophils, and type 2 innate lymphoid cells, participate in the perpetuation of the inflammatory microenvironment (3). Environmental and systemic factors, such as smoking, hepatitis B or C infection, hypertension, cardiovascular disease, asthma, and nephrotic syndrome, have also been proposed as potential contributors to KD onset (3).

### 4.3 Clinical manifestations

Lesions predominantly occur in the head and neck regions, commonly presenting as painless facial swelling, salivary gland involvement, and regional lymphadenopathy (4). However, involvement of the orbit, oral cavity, axilla, groin, extremities, and trunk has also been reported (12). Uncommon dermatological manifestations include generalized pruritus, eczema, and prurigo nodularis; systemic constitutional symptoms are generally absent. The incidence of pruritus in KD patients increases with age (10). Approximately 20% of KD patients develop renal disease, including minimal change disease, mesangial proliferative glomerulonephritis, and membranous nephropathy. Among those with renal involvement, around 60% to 80% present with nephrotic syndrome (5).

Among the 12 reviewed cases of extremity-predominant KD, the chief complaint was uniformly a painless, firm, non-tender mass, occasionally accompanied by regional lymphadenopathy. A single case case (1/12) presented primarily as pruritic plaques on the lower limb, a manifestation potentially related to cutaneous eosinophilic infiltration (13).

### 4.4 Diagnosis and differential diagnosis

Diagnosis of KD requires an integration of clinical, laboratory, and pathological findings. In the majority of cases, laboratory studies reveal peripheral eosinophilia and markedly elevated serum IgE levels (>90%) (10). Higher absolute eosinophil counts have been associated with poorer prognosis (14). Nonetheless, the present cases and at least one additional reviewed case (15) demonstrate that KD may occur without these serological hallmarks. This atypical presentation is plausibly explained by the localized and early-stage nature of the disease: when lesions are small and confined to a single site, the Th2-mediated immune activation may remain spatially restricted, insufficient to generate detectable systemic eosinophilia or IgE elevation. In the present case, neither peripheral eosinophilia nor elevated serum IgE was detected, a rare but previously documented presentation of KD (10, 15). The absence of peripheral eosinophilia and elevated IgE should therefore not exclude KD from the differential diagnosis when histopathological features are characteristic.

Imaging studies, including ultrasound, CT, and MRI, often demonstrate ill-defined subcutaneous or glandular masses. which may be solitary or multiple, with heterogeneous contrast enhancement (6, 7, 16). In the extremities, KD masses typically range from 2 cm to 7 cm in diameter. On CT, soft tissue masses often appear isodense. On MRI, they are isointense or mildly hyperintense on T1 and T2/STIR sequences, often surrounded by tissue edema with localized thinning of subcutaneous fat.

Histopathological examination remains the gold standard for KD. Essential features include follicular lymphoid hyperplasia with reactive germinal center formation, dense eosinophilic infiltration within follicles and interfollicular regions, eosinophilic microabscess formation, and proliferation of postcapillary high endothelial venules (3, 6). In later-stage disease, progressive fibrosis or even hyalinization may supervene. Among the 12 reviewed extremity cases, a broadly consistent histopathological pattern was identified: lymphoid follicles with mature lymphocyte infiltration were apparent at low magnification; germinal centers were identified in 2/12 cases; high-power examination revealed diffuse eosinophilic infiltration with microabscess formation; and small vessel proliferation was noted in 8/12 cases. In the present case, the vascular markers CD31 and CD34 served to confirm intratumoral small vessel proliferation, a recognized histological feature of KD. These markers were not intended to—and do not—independently confirm KD; the diagnosis was established on the basis of the integrated clinicopathological constellation described above.

### 4.5 Differential diagnosis

KD affecting the extremitiesmust be differentiated from a spectrum of neoplastic and inflammatory conditions. Among these, the distinction from angiolymphoid hyperplasia with eosinophilia (ALHE) warrants particular emphasis, given the clinical and histological overlap between the two entities.

ALHE typically presents in young-to-middle-aged individuals of any ethnicity as red-to-brown papules or nodules predominantly in the head and neck region, and—unlike KD—serum IgE is typically normal (17, 18). Histologically, the key distinguishing feature of ALHE is the presence of irregular vascular proliferation lined by epithelioid or “hobnail” endothelial cells with cytoplasmic vacuolation, a feature absent in KD. In contrast, KD is characterized by well-formed germinal centers, a more prominent and diffuse eosinophilic infiltrate, and absence of endothelial atypia (18). In the present case, the absence of epithelioid endothelial changes, the presence of well-formed follicular hyperplasia, and the pattern of dense eosinophilic microabscess formation collectively support a diagnosis of KD over ALHE. The normal IgE and eosinophil count, while potentially creating diagnostic ambiguity, do not preclude KD in the appropriate clinicopathological context.

Lipoma is distinguished by its composition of mature adipocytes without lymphoid or eosinophilic elements (19, 20). Eosinophilic granulomatosis with polyangiitis (EGPA) may be excluded in the present case by the absence of asthma, pulmonary infiltrates, ANCA positivity, and medium-vessel necrotizing vasculitis (21, 22) (Table 2).

**Table 2:** Differential diagnoses of Kimura disease affecting the extremities.

Feature	KD	Lipoma	EGPA	ALHE
Epidemiology	Predominantly affects Asian males aged 20–30 years.	Can occur at any age; more common in adults.	More frequent in middle–aged populations in Europe and North America; no clear gender predominance.	Typically presents between ages 20–40, with no gender predominance.
Clinical Features	Painless subcutaneous soft tissue masses or lymphadenopathy, most often in the head and neck region.	Slow–growing, painless subcutaneous nodules commonly on the neck, shoulders, back, and thighs.	Asthma; small–vessel vasculitis (often ANCA–positive); eosinophil–mediated manifestations such as eosinophilic myocarditis and pulmonary infiltrates.	Red–to–brown papules or nodules, predominantly localized to the head and neck.
Laboratory Findings	Peripheral eosinophilia and elevated serum IgE (>90% of cases); may be normal in early or localized disease.	Non–specific.	Peripheral eosinophilia; positive ANCA (especially MPO–ANCA).	Peripheral eosinophilia; IgE typically normal in classic ALHE, though elevated IgE has been reported.
Histopathology	Follicular hyperplasia, germinal centers, eosinophilic infiltrates, and small vessel proliferation. No endothelial atypia.	Mature adipocytes in irregularly sized lobules separated by fibrous connective tissue; no eosinophilic infiltration.	Necrotizing inflammation of small–to–medium vessels with abundant eosinophils; no prominent germinal center formation.	Irregular vascular proliferation with prominent epithelioid/hobnail endothelial cells; lacks well–formed germinal centers; fewer eosinophils than KD.
Treatment	Surgery, corticosteroids, radiotherapy, biologics targeting Th2 pathway.	Surgical excision.	Corticosteroids, immunosuppressants, biologics (mepolizumab, benralizumab).	Surgery, corticosteroids, biologics (dupilumab).
References	(3), (31)	(19), (20)	(21), (22)	(17), (18)

### 4.6 Management and prognosis

No universally accepted treatment protocol exists for KD. Available interventions include surgical excision or radiotherapy for localized lesions, and systemic corticosteroids or immunosuppressive agents (cyclosporine, methotrexate, leflunomide) for more extensive or recurrent disease (4, 23). Low-dose long-term steroid maintenance has demonstrated efficacy in selected patients (24). More recently, biologic agents targeting the Th2 immune axis—including dupilumab (anti-IL-4Rα), mepolizumab and benralizumab (anti-IL-5), and omalizumab (anti-IgE)—have shown promising results in refractory cases (3).

A critical therapeutic distinction warrants clarification: in the present case, the patient declined specific disease-modifying immunosuppressive agents (cyclosporine, methotrexate) due to concerns about their toxicity profile and the perceived mildness of his disease. He instead accepted long-term low-dose glucocorticoid therapy (prednisolone 5 mg/day). Although glucocorticoids possess immunosuppressive properties at higher doses, low-dose maintenance regimens are widely used in chronic inflammatory conditions primarily for their anti-inflammatory rather than lymphocyte-depleting effects. This distinction is clinically meaningful: the patient's reluctance was directed toward cytotoxic or T-cell-targeting immunosuppressants, not corticosteroids *per se*.

Surgery remains the primary treatment for extremity KD. However, lesion margins may be ill-defined in infiltrative cases, and positive surgical margins are associated with higher recurrence rates (8). The present patient achieved a 16-month recurrence-free outcome following complete surgical excision combined with low-dose prednisolone maintenance. However, this outcome cannot be attributed solely to glucocorticoid therapy, given that the lesion was small, solitary, and excised with confirmed [negative/close] surgical margins. In cases of complete surgical excision of small, serologically quiescent KD lesions, the necessity of routine postoperative glucocorticoid maintenance has not been established by prospective data, and the decision to continue treatment should be individualized, weighing the chronic inflammatory burden of KD against the cumulative risks of prolonged low-dose steroid exposure. Extended radiological follow-up beyond 16 months remains warranted.

## 5 Conclusion

KD is a rare chronic inflammatory disorder with a well-established predilection for the head and neck region. The present case demonstrates that KD may manifest exclusively as a localized, painless soft tissue mass of the lower extremity—in this instance, the right pretibial region—in the complete absence of peripheral eosinophilia and elevated serum IgE. This atypical presentation underscores a critical diagnostic principle: KD should remain in the differential diagnosis of extremity soft tissue masses even when classical serological hallmarks are absent, and histopathological examination is indispensable for establishing the diagnosis. Rigorous pathological analysis—comprising the identification of follicular hyperplasia, dense eosinophilic infiltration with microabscess formation, small vessel proliferation, and careful exclusion of ALHE, lymphoma, and IgG4-related disease—is essential. Surgical excision with confirmed negative margins represents the cornerstone of treatment for localized extremity KD. The role of adjuvant low-dose glucocorticoid maintenance in preventing local recurrence following complete excision of small, serologically quiescent lesions requires further prospective evaluation. Given the known chronic and relapsing nature of KD, long-term—potentially lifelong—clinical and radiological surveillance is recommended for all patients, regardless of initial treatment modality.

## Data Availability

The original contributions presented in the study are included in the article/supplementary material, further inquiries can be directed to the corresponding author.
